# Beyond Analgesics: Physical Activity as a Potential Approach to Pain-Related Outcomes in Older Adults—Preliminary Evidence

**DOI:** 10.3390/jcm15093498

**Published:** 2026-05-02

**Authors:** Aleksandra Budzisz

**Affiliations:** 1 Pain Research Group, Institute of Psychology, Jagiellonian University, 30-060 Krakow, Poland; 2 Academy of Physical Education in Katowice, Humanistic Foundations of Physical Culture, 40-065 Katowice, Poland; a.budzisz@awf.katowice.pl

**Keywords:** pain, pain awareness, passive awareness, interoception, yoga, older adults, physical activity

## Abstract

**Background/Objectives**: With an increasing number of older adults remaining physically active into later life, there is a growing need to understand how they manage pain and stress without relying on pharmacological treatment. Although regular physical activity supports functional independence and psychological resilience, many active older adults still experience fluctuating pain or stress. However, they often prefer non-pharmacological strategies and avoid analgesics, even when experiencing pain. Yoga interventions are widely used to address both physical and psychological components of health across diverse populations. **Methods**: Twenty-three adults aged ≥65 years participated in a once-weekly, 60 min yoga program. Pain intensity (VAS), pain vigilance and passive awareness (PVAQ), coping strategies (CSQ), and depression, anxiety, and stress (DASS-21) were assessed pre- and post-intervention. Repeated-measures ANOVA and correlational analyses were conducted. Exploratory moderation analyses examined whether individual characteristics (physical activity, age, and yoga experience) influenced associations between changes in pain-related variables. **Results**: After participation in a 7-week yoga program, significant differences were observed: perceived stress decreased, and passive pain awareness increased. No significant changes occurred in pain intensity, fear, depression (although a decrease was observed), or coping strategies, although participants predominantly used adaptive coping at both time points. Moderation analyses showed that physical activity buffered the association between increased passive pain awareness and heightened pain, whereas age and prior yoga experience strengthened this association. **Conclusions**: Even in physically active older adults, yoga participation was associated with changes in passive pain awareness and reduced stress. However, increases in passive pain awareness may differentially influence pain depending on age, physical activity level, and previous yoga experience.

## 1. Introduction

The proportion of older adults aged 65 years and above is increasing worldwide, creating a need to address the health and well-being of this growing population. Although age alone does not necessarily determine health decline, epidemiological data indicate that a high percentage of individuals over 65 experience chronic pain and multiple comorbidities [1]. Women, who tend to outlive men, represent the majority of this demographic and are particularly affected by musculoskeletal pain and functional limitations [2,3,4,5].

Maintaining physical independence and functional capacity is a primary determinant of healthy aging. Regular physical activity supports blood pressure regulation, muscular strength, flexibility, and balance while reducing the risk of metabolic and cardiovascular diseases [6]. In modern societies, the availability of tailored exercise programs for older adults continues to grow, reflecting an increasing interest in non-pharmacological approaches that promote self-management of health and pain. Such interventions are particularly valuable in this population because, unlike pharmacological treatments, they are associated with fewer side effects and may enhance both physical and psychological resilience.

Among mind–body practices, yoga has gained popularity due to its dual focus on the body and the mind. The growing research interest is reflected in the sharp rise in yoga-related publications since 2005, emphasizing its perceived potential as a complementary health intervention. Through the combination of postural exercises (asanas), breathing control (pranayama), and focused attention, yoga integrates movement and mindfulness, providing benefits for physical fitness and emotional balance [7,8], as well as for physiological parameters, including reductions in total and LDL cholesterol, plasma insulin levels, and insulin resistance [9,10], among older adults. Numerous studies have demonstrated that yoga can improve flexibility, muscle strength, and posture [8,9,10] while also lowering stress, anxiety, and depressive symptoms [11,12]. Meta-analyses confirm that yoga produces small-to-moderate improvements in pain intensity and disability across various chronic pain conditions [13,14,15,16].

As decreased pain is one of the documented effects of yoga, the biopsychosocial model of pain suggests that cognitive and attentional processes play a central role in shaping pain perception [17]. While numerous trials have evaluated yoga’s effects on single outcomes such as pain intensity, depression, or anxiety, relatively few have examined multidimensional profiles underlying pain processing—including sensitivity to pain (pain vigilance), awareness of pain-related sensations (pain awareness), and the strategies individuals use to cope with pain (pain coping strategies). Assessing these domains simultaneously is important in order to capture a comprehensive picture of pain-related experiences, as reflected in the definition of pain proposed by the International Association for the Study of Pain [17], which emphasizes the sensory, emotional, cognitive, and behavioral dimensions of pain. Addressing these interrelated components may therefore provide a more integrative understanding of how yoga interventions interact with pain-regulatory processes in aging populations.

However, despite the expanding evidence base, several gaps remain. The majority of randomized controlled trials (RCTs) have focused on sedentary or clinically limited populations, while there is a need for further research into the effects of yoga interventions among older adults who are already physically active. These individuals may differ from sedentary peers in terms of baseline physical activity and related physiological characteristics (e.g., muscle mass and metabolic profile), which may influence pain processing, suggesting that the effects of yoga may operate differently in this group.

Within this framework, baseline and habitual physical activity may contribute as a secondary moderator to how individuals respond to a yoga intervention. Yoga, as demonstrated in previous studies, may modulate cognitive–attentional mechanisms by passive observation and promoting more mindful, flexible forms of coping [11,16].

The primary aim of this study was to evaluate changes in response to a 7-week yoga program in pain-related and psychological variables in physically active older adults. Participants were evaluated before and after the yoga intervention on pain intensity (VAS), pain vigilance (PVAQ), depression, anxiety, and stress (DASS-21), and pain coping strategies (CSQ). Based on prior literature and preregistered assumptions, three hypotheses were formulated:

**H1.**  *Participation in the yoga intervention will be associated with changes in at least one pain-related variable.*

**H2.**  *Changes in attentional or awareness-based pain variables may be associated with changes in pain intensity.*

**H3.**  *The magnitude or direction of these associations may vary due to individual differences (e.g., physical activity, age, and yoga experience).*

Individual characteristics were therefore treated as potential moderators of change, rather than primary outcomes of interest.

## 2. Materials and Methods

### 2.1. Study Design

This study employed a within-subject design to examine the psychological and perceptual changes over time following participation in a 7-week yoga program in physically active older adults. Given the single-group design, any observed changes are interpreted as within-subject associations over time. Data were collected in a regional urban area in Poland between September and November 2025. All procedures were conducted in accordance with the Declaration of Helsinki, and written informed consent was obtained from each participant prior to enrollment. The study protocol was preregistered on the Open Science Framework (OSF): https://osf.io/ykavx (accessed on 13 March 2026).

### 2.2. Participants

Participants were recruited through local activity centers for older adults. However, the program evaluated in this study consisted only of yoga sessions. Eligibility criteria included being aged 65 years or older, engaging in at least one structured group physical activity per week, and willingness to attend a yoga program. Participants were required to have sufficient cognitive and reading ability to complete self-report questionnaires independently. Individuals with acute musculoskeletal injuries, uncontrolled cardiovascular conditions, or cognitive impairments preventing safe participation were excluded.

A total of 23 participants (21 women, 2 men) were enrolled. The required sample size was calculated a priori using G*Power 3.1 for a repeated-measures design comparing pre- and post-intervention outcomes (two-tailed test, α = 0.05, power = 0.80). Based on previous evidence suggesting a moderate effect of yoga on pain outcomes (SMD = −0.74), a conservative effect size of f = 0.35 was used for the a priori power analysis [13]. The analysis indicated that 19 participants were sufficient to detect significant within-subject differences, suggesting that the final sample size was adequate for detecting pre–post changes within individuals. This estimation was consistent with the preregistered assumptions.

### 2.3. Seven-Week Yoga Program

The program lasted seven weeks, consisting of one 60 min group session per week. Each session integrated three core components: (1) physical postures, (2) breathing exercises, and (3) guided relaxation. Classes were delivered by a certified yoga instructor with experience in teaching older adults. Modifications and use of supportive props were provided to accommodate varying mobility levels and to ensure safety. The sessions were conducted in a gymnasium providing appropriate infrastructure and sufficient space for comfortable exercise. The duration of the program was selected based on previous studies indicating that similar short-term yoga programs are sufficient to detect changes in psychological and pain-related outcomes [13,14].

### 2.4. Measures

Self-report instruments were administered at baseline and after the 7-week program to assess pain processing, including pain perception, passive awareness, vigilance, coping mechanisms, and emotional functioning (depression, fear and stress). All questionnaires were administered in a paper-and-pencil format and completed individually by participants. Assistance was provided when needed to ensure understanding of the items, without influencing responses.

Pain intensity was measured using a 100 mm Visual Analog Scale (VAS), where participants indicated their average level of pain experienced on the day of assessment, during the past week, the past month, and the past six months.

The Pain Vigilance and Awareness Questionnaire (PVAQ) [18,19] was used to assess the degree of attention individuals allocate to pain-related sensations. The scale assesses two distinct aspects of attentional focus on pain. Pain vigilance reflects a heightened orientation marked by hyper-attention and excessive monitoring. In contrast, pain awareness pertains to the ability to notice and observe pain as it appears and diminishes over time.

Cognitive and behavioral coping responses to pain were assessed using the Coping Strategies Questionnaire (CSQ) [20,21]. This measure captures how individuals cognitively reinterpret or behaviorally manage pain through eight subscales, including catastrophizing, praying or hoping, reinterpreting pain sensations, ignoring sensations, increasing behavioral activity, and diverting attention. Higher scores on adaptive strategies (e.g., reinterpretation, distraction) indicate more constructive coping, whereas elevated catastrophizing scores suggest maladaptive patterns.

Depression, anxiety, and stress were assessed with the Depression, Anxiety, and Stress Scale-21 (DASS-21) [22,23], a 21-item measure evaluating three interrelated negative affective states: depression (low mood, anhedonia), anxiety (autonomic arousal, tension), and stress (irritability, difficulty relaxing). Participants rated the frequency of each symptom over the past week on a 4-point scale, with higher scores indicating greater emotional distress.

Demographic variables, such as age, weight, height, yoga experience, comorbidities, and analgesic use, were recorded.

To capture participants’ engagement in physical activity, two separate indices were calculated: one representing structured non-yoga activities (e.g., dance, aerobics, Pilates) and another reflecting yoga practice specifically. The perceived intensity of each activity type was assessed using the Borg CR10 Scale, a validated measure of subjective exertion ranging from 0 (no exertion) to 10 (maximum effort). For both activity types, a physical activity intensity index (PAII) was computed using the formula: PAII = frequency of activity (sessions/week) × session duration (minutes) × perceived exertion (CR10 rating). The first index (PAII-AF) reflected the overall physical activity load from non-yoga movement practices, whereas the second index (PAII-yoga) quantified the intensity and volume of yoga participation during the intervention. This dual-index approach provided a nuanced assessment of participants’ physical activity patterns both within and outside the yoga program.

Additionally, participants indicated whether they met the World Health Organization recommendation of ≥150 min of moderate-intensity physical activity per week by selecting “yes” or “no”.

### 2.5. Statistical Analysis

Analyses were performed using IBM SPSS Statistics (Version 29). First, descriptive statistics (means, standard deviations, frequencies, and percentages) were calculated to characterize the sample in terms of demographic, clinical, and activity-related variables. All analyses followed a preregistered analytical framework focusing on (1) within-subject change, (2) associations between change scores, and (3) their moderation by individual characteristics. Participants who did not attend all intervention sessions or did not complete either the pre- or post-intervention assessment were excluded from the analyses.

To evaluate changes associated with the yoga intervention, repeated-measures analyses were conducted. Depending on the distributional properties of each variable, either repeated-measures ANOVA (with Greenhouse–Geisser correction when sphericity assumptions were violated) or non-parametric equivalents were used. For all ANOVA models, eta squared (η^2^) was reported as the effect size. Ninety-five percent confidence intervals were estimated using bootstrapping procedures. Post hoc pairwise comparisons were adjusted using the Bonferroni correction.

Associations between changes in key outcomes (Δ scores) and individual characteristics were examined using Spearman’s rank-order correlations due to the non-normal distribution of several variables.

To examine whether individual differences were associated with changes in key variables, a series of moderation analyses was conducted using Model 1 of the PROCESS macro for SPSS (version 5.0). Moderators tested included baseline physical activity level (PAII-AF), age, and yoga experience. For each model, unstandardized coefficients, standardized effects (b), significance levels, explained variance (R^2^), and changes in explained variance (ΔR^2^) were reported. Conditional effects were further evaluated using the Johnson–Neyman technique to identify regions of significance.

All statistical tests were two-tailed, with significance set at *p* < 0.05.

## 3. Results

### 3.1. Participant Characteristics

The mean age of participants was 69 years (range: 61–79), and the sample consisted predominantly of women. Most individuals had a BMI within the normative range (M = 20.71, SD = 3.33). The majority had a higher education and were retired, and more than half were married. Most participants reported a monthly income between 2000 and 5999 PLN, although some declined to provide this information (n = 7).

The duration of chronic pain ranged from 1 to over 5 years, with almost half of the participants reporting pain lasting more than 5 years. Over half of the participants had a formal medical diagnosis (most commonly osteoarthritis, followed by rheumatoid arthritis and other conditions, e.g., neuropathy, osteoporosis, or Parkinson’s disease). In addition, a substantial proportion of participants reported chronic non-specific musculoskeletal pain without a formal diagnosis.

Patterns of analgesic use indicated a general preference for non-pharmacological pain management. Furthermore, the majority reported avoiding analgesics even during episodes of pain, reinforcing the tendency toward non-pharmacological strategies.

Participants were already physically active prior to the study. Most engaged in at least one organized weekly activity in addition to yoga, and a substantial proportion reported two or more distinct forms of structured exercise per week. Yoga experience varied, with nearly half reporting 2–5 years of practice.

Participants rated their typical non-yoga physical activity as moderately intense (mean exertion ≈ 6.5/10), whereas yoga sessions were rated as having a lower intensity (≈4.5/10). Detailed demographic and background characteristics are presented in Table 1.

### 3.2. Primary Outcomes

A repeated-measures ANOVA with Greenhouse–Geisser correction revealed a statistically significant effect of the yoga program on passive pain awareness, as measured by the PVAQ, with a large effect size, F(1.22, 384.54) = 4.88, *p* = 0.038, η^2^ = 0.182. Pairwise comparisons using Bonferroni correction showed a significant increase in pain awareness following the yoga intervention (M = 22.44, SD = 1.79), compared to baseline levels (M = 16.65, SD = 2.60; *p* = 0.038).

Similarly, a repeated-measures ANOVA with Greenhouse–Geisser correction indicated a statistically significant effect of the yoga intervention on stress, as measured by the DASS stress subscale, also with a large effect size, F(1.22, 384.54) = 7.15, *p* = 0.014, η^2^ = 0.245. Pairwise comparisons with Bonferroni adjustment showed a significant reduction in perceived stress after the yoga intervention (M = 3.61, SD = 0.79), relative to pre-intervention levels (M = 5.65, SD = 0.85; *p* = 0.014).

Analyses revealed no statistically significant effects of the yoga intervention on pain-related variables (pain intensity, pain vigilance) or psychological functioning (fear, depression).

Also, no statistically significant differences were observed across the CSQ subscales. At the descriptive level, the most frequently endorsed coping strategies at baseline and post-intervention were coping self-statements and increased behavioral activity, whereas catastrophizing remained the least frequently used strategy. Detailed results of these analyses are presented in Table 2.

### 3.3. Correlational Findings

Spearman’s rank-order correlations (ρ) were computed to explore associations between change scores (Δ = time point 2 − time point 1) in outcome variables—including pain coping strategies, pain vigilance, fear, depression, stress, and pain intensity—and participant characteristics such as analgesic use, physical activity engagement (defined as meeting the guideline of >150 min of moderate physical activity in the past 7 days), length of yoga experience, the general physical activity index (PAII-AF), and the yoga-specific activity index (PAII-yoga). This approach allowed us to identify individual factors associated with psychological and behavioral responses to the yoga intervention.

The PAII-yoga index was significantly and positively correlated with several pain coping strategies: distraction (ρ = 0.39, *p* = 0.03), reinterpreting pain sensations (ρ = 0.46, *p* = 0.01), ignoring pain sensations (ρ = 0.53, *p* = 0.01), praying/hoping (ρ = 0.45, *p* = 0.02), self-statements (ρ = 0.56, *p* < 0.01), and increased behavioral activity (ρ = 0.48, *p* = 0.01). Additionally, PAII-yoga was positively associated with stress (ρ = 0.39, *p* = 0.04) and negatively associated with fear (ρ = −0.36, *p* = 0.04). These findings suggest that greater engagement in yoga was linked to both active coping styles and more intense emotional responses.

The length of yoga experience was negatively correlated with the use of pain reinterpretation (ρ = −0.35, *p* = 0.05) and ignoring pain sensations (ρ = −0.59, *p* = 0.00) and positively associated with the perceived ability to control pain (ρ = 0.35, *p* = 0.05). This may indicate that longer-term practitioners rely less on passive strategies and feel more in control of their pain. Yoga experience was also negatively associated with general physical activity (PAII-AF) (ρ = −0.54, *p* = 0.00), indicating that more experienced participants tended to have lower levels of non-yoga physical activity. The self-reported adherence to physical activity guidelines (i.e., more than 150 min of moderate activity per week) was negatively associated with changes in pain intensity (ρ = −0.46, *p* = 0.02), suggesting that participants who met the recommended level of activity experienced greater pain reduction.

Change in pain intensity (Δpain) was positively associated with changes in perceived stress (ρ = 0.38, *p* = 0.04), passive pain awareness (ρ = 0.47, *p* = 0.01), and active pain vigilance (ρ = 0.42, *p* = 0.02), indicating that increases in these psychological variables were related to changes in pain. This suggests that heightened attentional focus on pain and stress may contribute to less favorable pain outcomes.

Finally, analgesic use was negatively associated with depressive symptoms (ρ = −0.44, *p* = 0.02), suggesting that participants who used pain medication reported lower levels of depression as measured by the DASS. This may reflect the mood-regulating effect of effective pharmacological pain management. Correlational results are presented in Table 3.

Due to the absence of a statistically significant effect of the yoga intervention on pain intensity but a notable increase in passive pain awareness, an exploratory moderation analysis was conducted to investigate whether changes in passive awareness were associated with changes in pain intensity. Based on theoretical frameworks linking attentional focus, physical engagement, and pain modulation, three variables were examined as potential mediators: overall physical activity level (PAII-AF), length of yoga experience, and age. These factors were selected because regular physical activity and long-term exposure to mind–body practices like yoga have been shown to influence both pain perception and attentional processes. In addition, age was considered a moderator, given its established role in shaping both pain sensitivity and attentional regulation in older adults. This analysis aimed to clarify whether the observed increase in pain awareness contributed to pain outcomes indirectly through behavioral or demographic pathways.

### 3.4. Moderation Analysis

#### 3.4.1. Physical Activity as a Moderator

**Figure 1:**
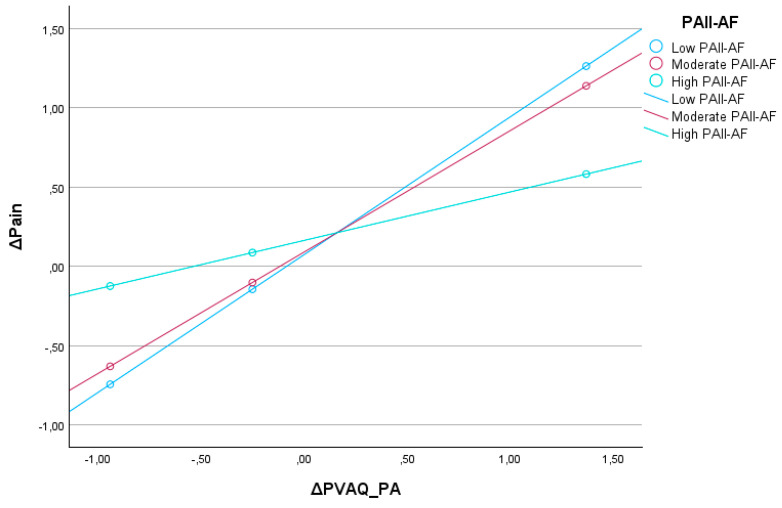
Physical activity as a moderator.

A moderation analysis was conducted to examine whether the overall physical activity level, indexed by the physical activity intensity index—aerobic fitness (PAII-AF), moderated the relationship between changes in passive pain awareness (ΔPVAQ_PA) and changes in pain intensity (ΔPain). The analysis was performed using Model 1 of the PROCESS macro [24]. The overall regression model was statistically significant, F(3,19) = 5.23, *p* = 0.008, accounting for 45.2% of the variance in the dependent variable (R^2^ = 0.45) (Figure 1).

Regarding main effects, an increase in passive pain awareness (ΔPVAQ_PA) was a significant predictor of increased pain intensity (b = 0.62, SE = 0.18, t = 3.42, *p* = 0.003), while the independent effect of physical activity level (PAII-AF) on ΔPain was not significant (b = 0.06, SE = 0.21, t = *p* = 0.78). Crucially, the interaction between ΔPVAQ_PA and PAII-AF was statistically significant (b = −0.38, SE = 0.15, t = −2.59, *p* = 0.018), indicating that the strength of the relationship between passive pain awareness and pain intensity varied depending on the level of physical activity. This interaction accounted for an additional 19.4% of variance in the outcome variable (ΔR^2^ = 0.194), F(1,19) = 6.73, *p* = 0.018. Conditional effects analysis and the Johnson–Neyman technique revealed that the effect of ΔPVAQ_PA on ΔPain was statistically significant only at low to moderate levels of physical activity (i.e., PAII-AF < 0.61). For example, among participants with low PAII-AF (−0.64), an increase in passive pain awareness was associated with a robust increase in pain intensity (b = 0.86, SE = 0.22, t = 3.92, *p* < 0.001, 95% CI [0.40, 1.33]). At moderate activity levels (PAII-AF = −0.39), the effect remained statistically significant (b = 0.76, SE = 0.20, t = 3.81, *p* = 0.001), whereas at higher levels of physical activity (PAII-AF = 0.83), the association between passive pain awareness and pain was no longer significant (b = 0.31, SE = 0.20, t = 1.54, *p* = 0.139).

These findings suggest that higher physical activity levels may play a protective, buffering role in the relationship between heightened passive pain awareness and increased pain perception. In other words, while increases in passive pain awareness generally predict increases in perceived pain intensity, this relationship weakens or disappears among individuals who are more physically active.

#### 3.4.2. Age as a Moderator

**Figure 2:**
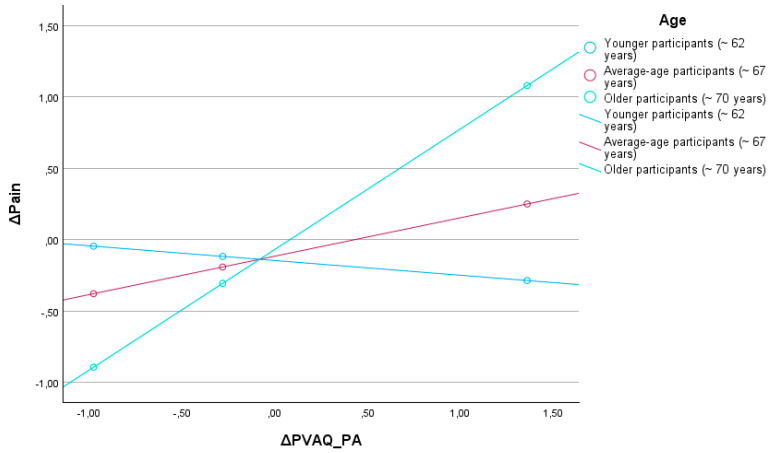
Age as a moderator.

A second moderation analysis was conducted to investigate whether age (Age) moderates the relationship between changes in passive pain awareness (ΔPVAQ_PA) and changes in pain intensity (ΔPain). The overall model was statistically significant, F(3,18) = 4.33, *p* = 0.018, accounting for 41.9% of the variance in the dependent variable (R^2^ = 0.42) (Figure 2).

The main effects were not statistically significant: the effect of ΔPVAQ_PA on pain was non-significant (b = 0.31, SE = 0.19, t = 1.56, *p* = 0.129), as was the effect of age (b = 0.01, SE = 0.04, t = 0.16, *p* = 0.876). However, the interaction term ΔPVAQ_PA × Age reached statistical significance (b = 0.08, SE = 0.03, t = 2.52, *p* = 0.0213), indicating that the impact of passive pain awareness on pain intensity differs depending on age. This interaction explained an additional 20.5% of variance in pain intensity (ΔR^2^ = 0.21), F(1,18) = 6.36, *p* = 0.021.

Conditional effects analysis showed that the effect of ΔPVAQ_PA on ΔPain became significant and stronger at higher age levels. Among older individuals (e.g., 74 years), the relationship between increased passive pain awareness and pain intensity was statistically significant (b = 0.85, SE = 0.26, t = 3.20, *p* = 0.005, 95% CI [0.29, 1.39]). In contrast, among younger participants (e.g., 64 years), the effect was non-significant (*p* = 0.712 and *p* = 0.193), indicating that increased passive awareness of pain did not translate into greater perceived pain in this subgroup.

Furthermore, the Johnson–Neyman technique identified that the effect of ΔPVAQ_PA on ΔPain became statistically significant at values of the moderator (mean-centered age) above 1.22. Given a sample mean age of 69 years (SD = 5.09), this corresponds to approximately 70.2 years. This finding suggests that the association between heightened passive pain awareness and increased pain intensity is present only among older individuals, while it does not reach statistical significance in younger participants.

In summary, the results indicate a significant interaction between passive pain awareness and age in predicting changes in pain intensity. Only among older participants was an increase in passive awareness associated with higher reported pain levels, which may suggest that age plays an important moderating role in cognitive-affective processes related to pain perception.

#### 3.4.3. Yoga Experience as a Moderator

**Figure 3:**
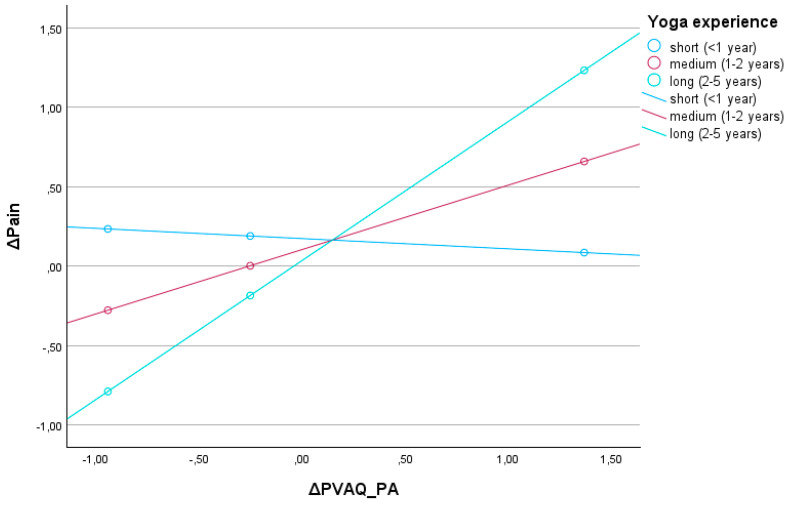
Yoga experience as a moderator.

A moderation analysis was conducted to examine whether yoga experience moderates the relationship between changes in passive pain awareness (ΔPVAQ_PA) and changes in pain intensity (ΔPain). The overall model was statistically significant, F(3,19) = 3.62, *p* = 0.032, explaining 36.4% of the variance in the dependent variable (R^2^ = 0.36) (Figure 3).

The main effect of ΔPVAQ_PA was statistically significant (b = 0.53, SE = 0.19, t = 2.76, *p* = 0.012), nor was yoga experience alone (b = −0.05, SE = 0.20, t = −0.26, *p* = 0.797). However, the interaction between ΔPVAQ_PA and yoga experience reached statistical significance (b = 0.33, SE = 0.16, t = 2.14, *p* = 0.045), indicating that the effect of changes in passive pain awareness on changes in pain intensity varied depending on the length of yoga practice.

Conditional effects analysis revealed that among participants with longer yoga experience (approximately 1 SD above the mean), increases in ΔPVAQ_PA were significantly associated with an increase in pain intensity (b = 0.88, SE = 0.27, t = 3.28, *p* = 0.004, 95% CI [0.32, 1.43]). In contrast, among participants with average or below-average yoga experience, the relationship between ΔPVAQ_PA and ΔPain was not statistically significant (b = −0.06, SE = 0.31, t = 0.20, *p* = 0.839, 95% CI [−0.72, 0.59]). Additionally, Johnson–Neyman analysis indicated that the effect of ΔPVAQ_PA on ΔPain became statistically significant at values of the moderator (yoga experience, standardized) above −0.36. This suggests that increased passive awareness of pain was significantly associated with increases in pain intensity only among individuals with moderate to long yoga experience, whereas this effect was not significant for participants with minimal yoga experience.

## 4. Discussion

The present study aimed to explore whether participation in a yoga program for physically active older adults would change pain processing, and whether this would be associated with individual characteristics. Although the yoga program did not lead to a statistically significant reduction in pain intensity, changes were observed in other pain-related variables, particularly in passive pain awareness and perceived stress. Specifically, passive pain awareness—defined as non-judgmental, mindful observation of pain—significantly increased post-intervention, while perceived stress levels significantly decreased. These findings are consistent with previous reports demonstrating that yoga and mind–body interventions can enhance interoceptive awareness and improve emotional regulation in older adults [25,26]. This may suggest that participation in yoga is associated with greater interoceptive awareness and improved stress regulation, even in an already active older population.

As the study focused on pain-related characteristics, the most frequently used pain coping strategy among participants was coping with self-statements, which showed a slight (but insignificant) increase following the yoga intervention. Increased behavioral activity was also commonly endorsed and remained at a comparable level post-intervention. These results may indicate that the coping strategies used by this group were largely adaptive and constructive. In contrast, catastrophizing—a less adaptive strategy—was the least frequently used. This predominance of adaptive coping is consistent with reports showing that physically active older adults tend to report lower anxiety [26], which may be considered in terms of catastrophizing. In qualitative studies, participants also refer to yoga as a tool for coping [27]. Such results may reflect participants’ high baseline level of physical activity, which could have already fostered adaptive coping styles, leaving limited room for further improvement through the yoga intervention. This stands in contrast to findings from randomized controlled trials involving less active populations, where more pronounced reductions in maladaptive coping strategies have been reported.

Highlighting the specificity of this cohort, it is noteworthy that correlation analyses showed that the pain coping strategies used by participants—with the exception of catastrophizing—were positively associated only with the yoga intensity index, a composite measure reflecting perceived exertion during the yoga sessions. This pattern may indicate that individuals who perceived the yoga intervention as more demanding were also more likely to engage in constructive coping strategies. Similar associations between perceived exertion, interoceptive focus, and psychological responses during yoga intervention have been noted in studies examining metabolic load and subjective intensity in yoga practice [9]. However, it should also be considered that perceiving the yoga sessions as more intense may reflect greater functional limitations or reduced physical capacity in some participants, which could influence both exertion ratings and their reliance on particular coping approaches.

A negative correlation between general physical activity (PAII-AF) and yoga experience was also observed, suggesting that older participants with longer yoga experience may engage less frequently in other forms of structured physical activity. This might reflect age-related physical limitations or a preferential focus on yoga as a primary form of movement practice.

Despite the absence of a significant change in pain intensity, the study provided valuable insight into the conditions under which passive pain awareness may influence pain outcomes. Specifically, moderation analyses revealed that the relationship between increases in passive pain awareness (ΔPVAQ-PA) and changes in pain intensity (ΔPain) was not uniform across participants but depended significantly on individual characteristics.

Age emerged as a significant moderator: increases in passive pain awareness were associated with higher pain intensity only among the older participants (approximately above 70 years). Age-related increases in interoceptive sensitivity and shifting neural processing in older adults have been described in previous work, providing a plausible mechanism for this effect [28]. This pattern may reflect reduced flexibility in attentional regulation, making bodily sensations more affectively salient. Yoga experience also moderated this relationship. For individuals with longer practice history, greater passive awareness was associated with higher pain intensity—a counterintuitive finding that may reflect heightened self-focus and increased sensitivity to bodily sensations in more experienced practitioners.

In contrast, the baseline physical activity level (PAII-AF) presented a potentially buffering effect. Among participants with higher overall physical activity, the link between increased passive awareness and increased pain intensity was attenuated and no longer significant. This aligns with findings that regular physical activity enhances attentional control and supports more neutral or adaptive interpretations of bodily sensations in older adults [6,29]. This finding suggests that regular physical activity may promote more adaptive attentional control or facilitate neutral interpretations of bodily cues (e.g., distinguishing discomfort associated with exertion from pain), thereby reducing the affective impact of increased awareness.

Although the change in pain was not statistically significant, the findings regarding individual traits—such as age, physical activity level, and body-based experience—may be associated with variability in how increased interoceptive awareness relates to pain perception. Among older or more experienced individuals, heightened body awareness may exacerbate pain, whereas physical activity appears to confer resilience against such effects.

Several limitations should be considered. The relatively small sample size, although sufficient for detecting within-subject changes, may limit power to detect smaller effects and to support more complex analyses; thus, correlational and moderation findings should be interpreted as exploratory. In addition, the duration of the yoga program (7 h total) and the absence of a control group limit conclusions about yoga program efficacy; therefore, the findings should be considered preliminary, indicating the need for further research to replicate the observed effects.

In summary, these findings indicate that planned interventions may be associated with outcomes that differ from initial expectations. They also underscore the importance of accounting for population-specific characteristics when designing and interpreting pain-related interventions.

No statistically significant changes were observed in pain intensity, depression, fear, or pain coping strategies. At the same time, significant changes were observed in perceived stress and passive pain awareness, while non-significant changes were found in pain coping strategies. Associations between pain-related variables and individual characteristics were further explored through correlation and moderation analyses, which showed that increased passive awareness of pain—often cultivated through yoga—may be considered as either an adaptive or maladaptive mechanism depending on individual factors. For physically active older adults, enhanced bodily awareness may support stress reduction and constructive coping; however, in certain subgroups, it may also intensify pain perception. Such heterogeneity is consistent with broader evidence showing that older adults vary widely in their responsiveness to yoga and mind–body interventions [14].

## Figures and Tables

**Table 1:** Participant characteristics (N = 23).

Variable	Category/Statistic	Value
Sex	Women	21 (91%)
	Men	2 (9%)
Age	Mean ± SD (range)	69.05 ± 5.09 (61–79)
Education	Secondary	6 (26%)
	Higher	17 (74%)
Employment status	Employed	1 (4%)
	Retired	22 (96%)
Monthly income (PLN)	2000–3999	5 (22%)
	4000–5999	9 (39%)
	6000–9999	1 (4%)
	≥10,000	1 (4%)
Marital status	Single	2 (9%)
	Married/partnered	15 (65%)
	Widowed	6 (26%)
Chronic condition duration	1–3 years	4 (17%)
	3–5 years	9 (40%)
	>5 years	10 (43%)
Type of diagnosis	non-specific musculoskeletal pain	10 (43%)
	Osteoarthritis	7 (30%)
	Rheumatoid arthritis	3 (13%)
	Neuropathy	1 (4%)
	Parkinson’s disease	1 (4%)
	Osteoporosis	1 (4%)
Analgesic use (general)	Yes	9 (39%)
	No	15 (65%)
Frequency of analgesic use	Daily	2 (9%)
	Several times a week	11 (48%)
	Several times a month	6 (26%)
	Several times a year	2 (9%)
	Never	2 (9%)
Analgesic use during pain episodes	Yes	10 (43%)
	No	13 (57%)
Anthropometrics	Body height (cm)	163.0 ± 7.88 (152–186)
	Body weight (kg)	67.73 ± 13.80 (48–105)
	BMI (kg/m^2^)	20.78 ± 10.84 (19–28)
	Average sleep time (min)	482.7 ± 95.3 (300–690)
Other physical activity sessions (per week)	0.00	1 (4%)
	1.00	14 (61%)
	2.00	4 (17%)
	3.00	4 (17%)
Perceived intensity of physical activity (0–10)	<6	6 (26%)
	6–7	10 (43%)
	>7	7 (30%)
Perceived intensity of yoga sessions (0–10)	3.00	4 (17%)
	4.00	6 (26%)
	5.00	6 (26%)
	6–9	7 (30%)
Yoga experience (years)	<1 year	5 (22%)
	1–2 years	7 (30%)
	2–5 years	11 (48%)

**Table 2:** Results of repeated-measures ANOVA examining pre–post changes across assessed pain-related variables.

Variable		M	SD	Min	Max	Mean Square	F	*p*	Partial Eta Square
PVAQ_PA_1		16.65	12.47	0.00	35.00				
PVAQ_PA_2	↑ *	22.43	8.57	1.00	35.00	384.54	4.88	0.04	0.18
PVAQ_AV_1		7.74	7.54	0.00	25.00				
PVAQ_AV_2	↑	10.48	6.97	0.00	25.00	86.28	3.18	0.09	0.13
Pain_1		24.23	23.95	0.00	65.00				
Pain_2	↓	22.76	22.40	0.00	73.00	24.84	0.19	0.76	0.01
CSQ_A_1		13.65	8.75	0.00	30.00				
CSQ_A_2	↓	13.22	7.85	0.00	27.00	2.17	0.19	0.67	0.01
CSQ_B_1		11.65	7.18	0.00	23.00				
CSQ_B_2	↓	9.22	7.99	0.00	27.00	68.17	2.25	0.15	0.09
CSQ_C_1		7.87	6.76	0.00	19.00				
CSQ_C_2	↓	6.91	7.02	0.00	22.00	10.52	0.63	0.44	0.02
CSQ_D_1		12.52	8.51	0.00	33.00				
CSQ_D_2	↑	15.87	8.45	0.00	33.00	128.89	2.46	0.13	0.10
CSQ_E_1		10.65	9.30	0.00	30.00				
CSQ_E_2	↑	11.22	10.54	0.00	36.00	3.67	0.12	0.74	0.01
CSQ_F_1		18.70	9.96	0.00	35.00				
CSQ_F_2	↑	20.00	8.88	0.00	36.00	19.55	0.43	0.52	0.02
CSQ_G_1		19.30	11.23	0.00	43.00				
CSQ_G_2	↓	18.17	7.74	0.00	30.00	14.70	0.30	0.59	0.01
CSQ_contr_1		4.00	1.45	0.00	6.00				
CSQ_contr_2	↑	4.13	1.42	0.00	6.00	0.09	0.09	0.77	0.00
DASS_D_1		2.70	2.64	0.00	8.00	3.13	1.15	0.30	0.05
DASS_D_2	↓	2.17	3.05	0.00	11.00				
DASS_F_1		3.13	3.68	0.00	14.00	6.28	3.27	0.08	0.13
DASS_F_2	↓	2.39	3.13	0.00	9.00				
DASS_S_1		5.57	4.08	0.00	14.00				
DASS_S_2	↓ *	3.61	3.82	0.00	11.00	44.02	7.15	0.01	0.25

Legend: M—mean, SD—standard deviation, PVAQ_PA—passive awareness, PVAQ_AV—active vigilance, CSQ_A—distraction, CSQ_B—reinterpretation of pain sensations, CSQ_C—catastrophizing, CSQ_D—ignoring pain sensations, CSQ_E—praying/hoping, CSQ_F—coping self-statements, CSQ_G—increased behavioral activity, CSQ_contr—perceived control over pain, DASS_D—depression, DASS_F—fear, DASS_S—stress, ↓—decrease in assessed variable, ↑—increase in assessed variable, * statistically significant change.

**Table 3:** Significant Spearman’s correlations between pain-related variables and individual characteristics.

Variable 1	Variable 2	Spearman’s ρ	*p*-Value
PAII_yoga	CSQ_A	0.39	0.03
PAII_yoga	CSQ_B	0.46	0.01
PAII_yoga	CSQ_D	0.53	0.01
PAII_yoga	CSQ_E	0.45	0.02
PAII_yoga	CSQ_F	0.56	*p* < 0.01
PAII_yoga	CSQ_G	0.48	0.01
PAII_yoga	ΔDASS_S	0.39	0.04
PAII_yoga	ΔDASS_F	−0.36	0.04
Yoga experience	CSQ_B	−0.35	0.05
Yoga experience	CSQ_D	−0.60	0.01
Yoga experience	CSQ_Contr	0.35	0.05
Yoga experience	PAII_AF	−0.54	0.00
ΔPain	ΔDASS_S	0.38	0.04
ΔPain	ΔPVAQ_PA	0.47	0.01
ΔPain	ΔPVAQ_AV	0.42	0.02
ΔPain	7 day 150 m	−0.46	0.02
ΔDASS_D	analgesic	−0.44	0.02

Legend: CSQ_A—distraction; CSQ_B—reinterpretation of pain sensations; CSQ_D—ignoring pain sensations; CSQ_E—praying/hoping; CSQ_F—coping self-statements; CSQ_G—increased behavioral activity; CSQ_Contr—perceived control over pain; DASS_S—stress; DASS_F—anxiety/fear; DASS_D—depression; PVAQ_PA—passive pain awareness; PVAQ_AV—active pain vigilance; PAII_yoga—physical activity intensity index for yoga (perceived exertion × frequency × duration); PAII_AF—physical activity intensity index for other structured activities; 7 day 150 min—meeting WHO recommendation of ≥150 min/week of moderate activity (0 = no, 1 = yes); analgesic—frequency of analgesic use.

## Data Availability

The data will be made available upon request to the corresponding author of this manuscript.

## Abbreviations

The following abbreviations are used in this manuscript:

CSQ	Pain Coping Strategies Questionnaire
CSQ_A	Distraction as a Pain Coping Strategy
CSQ_B	Reinterpretation of Pain Sensations as a Pain Coping Strategy
CSQ_C	Catastrophizing as a Pain Coping Strategy
CSQ_D	Ignoring Pain Sensations as a Pain Coping Strategy
CSQ_E	Praying/Hoping as a Pain Coping Strategy
CSQ_F	Coping Self-Statements as a Pain Coping Strategy
CSQ_G	Increased Behavioral Activity as a Pain Coping Strategy
CSQ_Contr	Perceived Control over Pain as a Pain Coping Strategy
PAII-AF	Physical Activity Intensity Index
PAII-Yoga	Yoga Index
PVAQ PA	Pain Vigilance Questionnaire: Passive Awareness Scale
PVAQ AV	Pain Vigilance Questionnaire: Active Vigilance Scale
DASS	Depression, Anxiety, and Stress Scale
